# His-Bundle Pacing in a Patient With Tricuspid and Mitral Prosthetic Valves Without Suitable Coronary Veins for Lead Placement

**DOI:** 10.1016/j.cjco.2021.03.012

**Published:** 2021-04-08

**Authors:** Javier Ramos-Maqueda, Mercedes Cabrera-Ramos, Nicole Southard, Adrián Riaño-Ondiviela, José Antonio Casasnovas-Lenguas, José Ramón Ruiz-Arroyo

**Affiliations:** a Department of Cardiology, Lozano Blesa Clinical University Hospital, Zaragoza, Spain; b Aragon Institute for Health Research (IIS Aragón), Zaragoza, Spain

## Abstract

Atrioventricular block in patients with a prosthetic tricuspid valve and a pacemaker with a dysfunctional epicardial lead is not uncommon. In such instances, coronary sinus lead placement is the preferred option, but it has a failure rate of 10%-15%. An atrial transseptal left ventricular lead placement has been proposed as an alternative, but this approach is not feasible in patients with a prosthetic mitral valve. This analysis represents the first reported case of His-bundle pacing from the atria in a patient with prosthetic tricuspid and mitral valves, with no suitable coronary veins for lead placement.

## Résumé

Le bloc auriculo-ventriculaire n'est pas rare chez les patients ayant reçu une valve tricuspide prothétique et porteurs d'un stimulateur cardiaque dont la sonde épicardique est dysfonctionnelle. Dans de tels cas, le positionnement de la sonde sur le sinus coronaire est l'option à privilégier, mais son taux d’échec varie entre 10 et 15 %. L'implantation de la sonde sur le ventricule gauche par la voie transsetale a été proposée à titre de solution de rechange, mais cette approche n'est pas envisageable chez les patients ayant reçu une valve mitrale prothétique. La présente analyse constitue le premier cas de stimulation du faisceau de His à partir des oreillettes chez un patient ayant reçu des valves tricuspides et mitrales prothétiques, en l'absence de veines coronaires se prêtant à l'implantation de la sonde.

## Case

A 74-year-old woman with recurrent episodes of syncope and heart failure symptoms was admitted to our hospital. She had a history of mitral and tricuspid valve replacement with mechanical prosthetic valves due to a rheumatic heart disease 30 years prior to admission. During that cardiac surgery, she developed atrioventricular block, and subsequently received a single-chamber pacemaker implant with an epicardial ventricular lead, owing to a history of permanent atrial fibrillation. In the most recent pacemaker reviews, she was found to have experienced a progressive rise in pacemaker lead impedance, as well as in the capture threshold (2500 ohms and 5.5 V at 1.00 ms). The presence of both a prosthetic tricuspid valve (PTV) and a prosthetic mitral valve made it impossible to place a new lead in the right or left ventricle (through a transseptal approach), as the latter would entrap the tilting discs of the valve. Therefore, the patient was referred for a coronary sinus lead placement, a procedure determined to be impossible in her case, due to the absence of suitable coronary veins.

**Figure 1:**
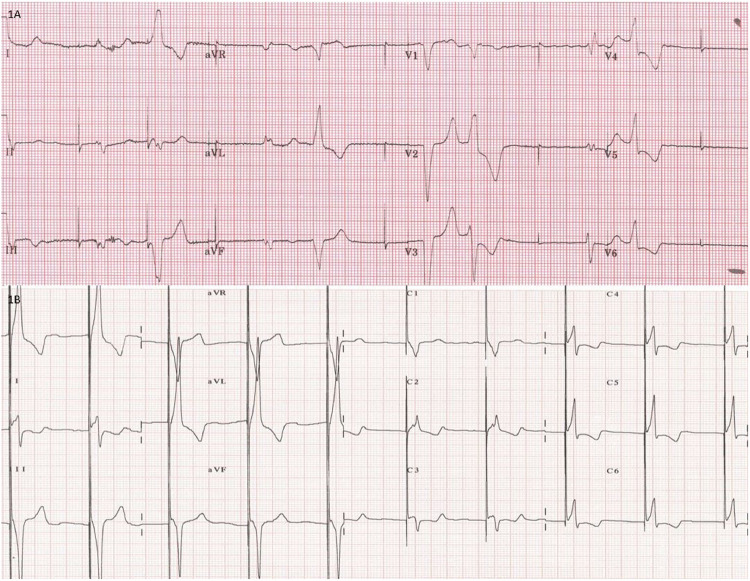
(**A**) Electrocardiogram showing capture failure. (**B**) Narrower QRS (128 ms) obtained after His-bundle pacing, with non-selective capture.

Upon her admission in our hospital, an electrocardiogram and an echocardiogram were performed. The electrocardiogram (Fig. 1A) showed obvious capture failure, which carries long ventricular pauses responsible for syncopal episodes and a wide intrinsic QRS complex (170 ms). The echocardiogram demonstrated biatrial enlargement, marked intraventricular dyssynchrony, and a left ventricular ejection fraction of 38%, with good performance of both prosthetic valves. Therefore, His-bundle pacing (HBP) from the right atrium was attempted, with the purpose of improving the QRS duration, and therefore the left ventricular ejection fraction.

**Figure 2:**
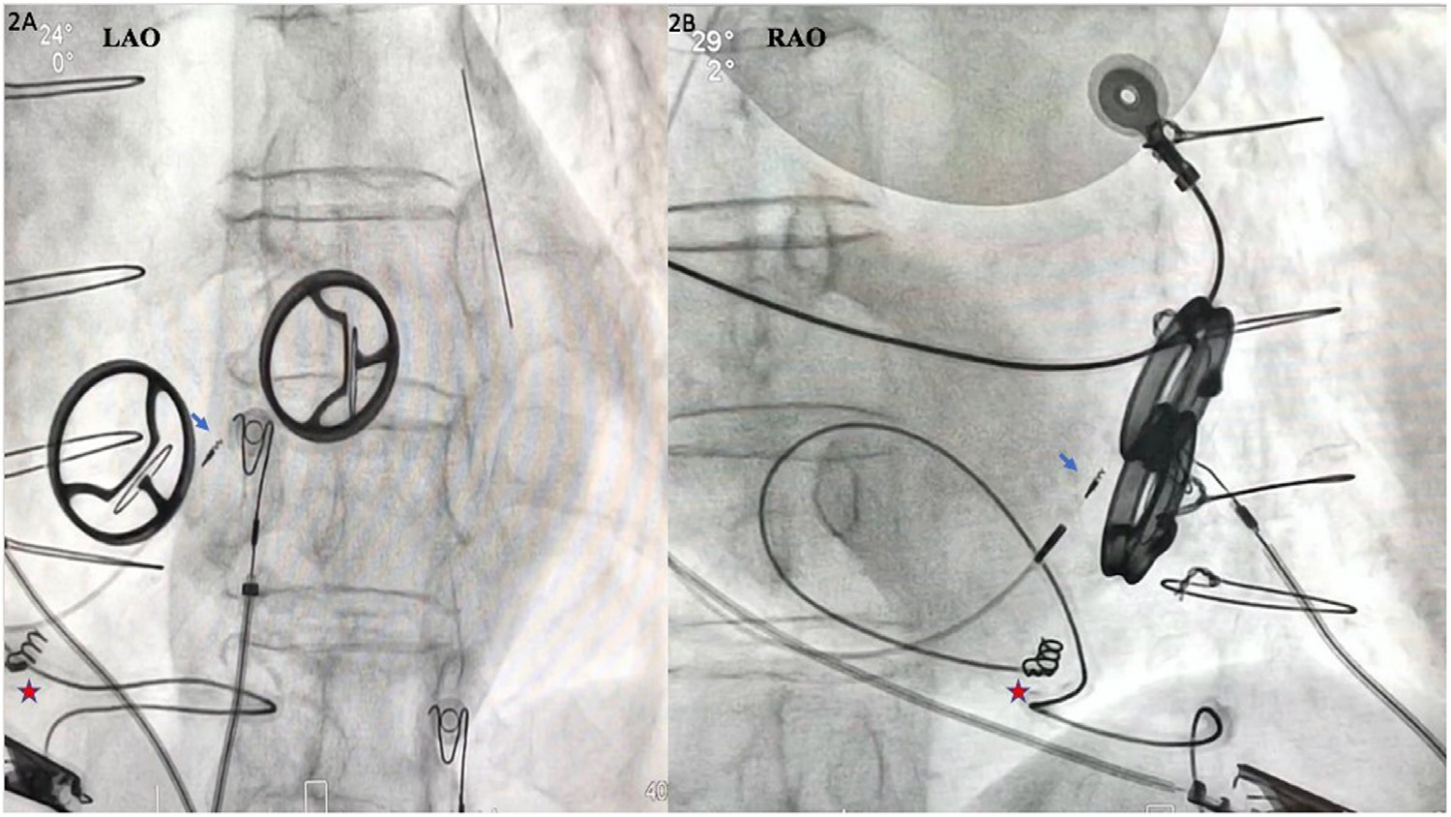
(**A**) Final lead fixation in left anterior oblique (LAO) view. (**B**) Final lead position in right anterior oblique (RAO) view. **Blue arrows** indicate His-bundle lead. **Red stars** indicate epicardial lead.

A deflectable sheath (C304model; Medtronic, Minneapolis, MN) was inserted, and through it a His Lead (3630 model, Medtronic). The sheath was torqued toward the annular mid-septum of the right atrium. His-bundle non-selective capture was obtained, and the lead was fixed (Fig. 2, A and B). The threshold was 3.6 V at 1.00 ms. A narrowed QRS complex was obtained (128 ms; Fig. 1B). During the follow-up, the patient exhibited improvement of the left ventricular ejection fraction to 50%, and the threshold remained stable.

## Discussion

We describe, for the first time, the feasibility and utility of HBP in a patient with no other option of intracardiac pacing because of 2 implanted mechanical prosthetic valves, unsuitable coronary veins, and dysfunction of the previous epicardial lead. In such instances, coronary sinus lead placement is the preferred option^1^^,^^2^; however, coronary sinus lead placement has a failure rate of 10%–15%. An atrial transseptal left ventricular lead placement has been proposed as an alternative,^3^ but this approach is not feasible in patients with a prosthetic mitral valve. Another option would be to replace the dysfunctional epicardial lead by cardiac surgery, a more invasive technique with more perioperative complications and significant morbidity.^4^

At this point, the possibility of performing physiological pacing from the His bundle appears to be the best option. HBP is an increasingly common approach because, together with left bundle branch pacing, it is the most physiological form of pacing.

HBP can be challenging in patients with a PTV.^5^ First is the concern of blocking the valve disks during the procedure with the sheath manipulation in this area. Second, although the atrial portion of the His bundle persists unaltered by the PTV, higher voltages could also be needed to capture the His, because of the closeness of the proximal His-bundle area to the PTV. However, we achieved a narrower QRS and a much less invasive procedure.

The risk of atrioventricular block after prosthetic valve surgery is not low. Because of the need for pacing in this group of patients, HBP offers a more physiological ventricular activation. Therefore, in this scenario, HBP emerges as a feasible alternative for cardiac pacing, anduse of the HBP approach should be considered.

## Novel Teaching Points

• HBP is a feasible alternative for cardiac pacing in patients with tricuspid and mitral mechanical valves.

## Funding Sources

The authors have no funding sources to declare.

## Disclosures

The authors have no conflicts of interest to disclose.
